# Impacto da Ablação com Campo Pulsado na de Fibrilação Atrial

**DOI:** 10.36660/abc.20240565

**Published:** 2024-10-24

**Authors:** Mauricio Scanavacca, Cristiano Pisani

**Affiliations:** 1 Hospital das Clínicas da Faculdade de Medicina da Universidade de São Paulo Instituto do Coração São Paulo SP Brasil Instituto do Coração do Hospital das Clínicas da Faculdade de Medicina da Universidade de São Paulo, São Paulo, SP – Brasil

**Keywords:** Fibrilação Atrial, Ablação por Cateter, Ablação por Campo Pulsado, Eletroporação

A fibrilação atrial (FA) é a arritmia cardíaca mais comum na prática clínica, com incidência crescente nos indivíduos mais idosos. É bem conhecido que a FA é um fator de risco substancial para eventos cardiovasculares, aumentando a morbidade e mortalidade entre os pacientes afetados, além de provocar redução da capacidade física, cognitiva e da qualidade de vida.^1-3^ O tratamento atual da FA baseia-se na modificação dos fatores clínicos agravantes, na introdução de anticoagulantes baseada na relação risco-benefício individualizada, de medicamentos para controle da frequência cardíaca e de antiarrítmicos para controle do ritmo, além da cardioversão elétrica naqueles com FA persistente.^1^

Nos pacientes refratários ou intolerantes ao tratamento clínico, a ablação por cateter com isolamento elétrico das veias pulmonares (VPs) vem sendo recomendada com entusiasmo crescente pelas diretrizes internacionais. De fato, os ajustes tecnológicos obtidos nos últimos anos na ablação por radiofrequência ponto a ponto e na crioablação por balão promoveram melhor efetividade do procedimento e maior segurança com ambas as tecnologias, demonstradas em numerosos trabalhos científicos comparativos recentes.^4,5^

A radiofrequência e a crioenergia são classificadas como energias térmicas, pois os seus efeitos terapêuticos resultam da liberação de calor na ablação com radiofrequência, ou do acentuado resfriamento durante a crioablação. Embora com ações antagônicas, ambas condições provocam necrose de coagulação bastante precisa do tecido atrial em contato com o cateter.^6^ A liberação da energia ao redor das VPs tem como objetivo isolar eletricamente os focos deflagradores das VPs do átrio esquerdo e assim evitar a ocorrência da FA. O grande desafio ao longo dos anos foi a obtenção de uma cicatriz circunferencial, transmural definitiva com resultados clínicos satisfatórios de longo prazo.^7,8^

Uma das principais limitações no uso dessas tecnologias é o ajuste preciso da entrega de energia. Esse ajuste deve garantir que a energia liberada provoque a lesão linear atrial necessária, periostial, contígua e transmural, sem causar estenose das VPs ou atingir tecidos adjacentes de forma indesejada, como lesões no nervo frênico, quando a energia é aplicada nas VPs direitas, ou lesões térmicas no esôfago, durante aplicações na região antral ou no isolamento da parede posterior do átrio esquerdo.^9,10^

A ablação por campo pulsado (PFA, do inglês – *pulsed field ablation*) é a mais nova forma de energia usada para isolamento elétrico das VPs que cria lesões no tecido miocárdico por meio de eletroporação irreversível, com efeito térmico mínimo nos tecidos afetados. Os estudos biofísicos e clínicos para o ajuste dessa energia em eletrofisiologia se iniciaram há aproximadamente 15 anos, com resultados clínicos em pacientes com FA nos últimos 5 anos.^11^

Essa nova energia para ablação caracteriza-se pela liberação de pulsos elétricos de alta energia pelo cateter, com duração de pulso extremamente curta, de poucos microssegundos. Esses pulsos provocam abertura de poros da membrana celular em contato com os eletrodos do cateter, gerando inundação de substâncias do líquido tissular no seu interior e sua inativação. Dependendo das características do pulso elétrico e energia do campo elétrico as alterações podem ser transitórias ou definitivas com morte celular mais intensa em tecidos específicos, característica interessante, apresentando seletividade na lesão tecidual.^11^

Os equipamentos que estão sendo introduzidos atualmente para uso clínicos foram testados e ajustados em avaliações experimentais e em estudos pré-clínicos e mostraram-se muito efetivos para provocar eletroporação irreversível no tecido atrial, sem afetar significativamente a parede das veias pulmonares, o nervo frênico justaposto às VPs direitas e ao esôfago.^11^

Vários ensaios clínicos demonstraram a eficácia do PFA em pacientes com FA paroxística refratária a medicamentos, com ausência de arritmias atriais variando de 66% a 87% um ano após o isolamento das VPs, com taxas de incidência variando de 0% a 2,5% para eventos adversos graves.^12-16^ Adicionalmente, apresentam menor tempo de procedimento e rápida curva de aprendizado. Esses aspectos técnicos tornam a ablação de FA mais acessível à população, envolvendo pacientes mais frágeis.

Um registro recém-publicado envolvendo 17.642 pacientes de centros europeus confirmou as observações de segurança preliminares, sem nenhum registro de casos de lesões esofágicas, estenose de veias pulmonares ou paralisia frênica, gerando grande entusiasmo mundial.^17^ Outra informação desse registro foi de que o número de complicações reduziu entre os 1.700 casos iniciais até os 17.000. Adicionalmente, um estudo randomizado recente de não inferioridade demonstrou equivalência no sucesso do procedimento quando comparou as formas convencionais de energias térmicas (radiofrequência e crio) à eletroporação irreversível.^18^

Apesar de segura, a utilização dessa tecnologia está associada a alguns efeitos adversos específicos desse tipo de energia. Um deles é o espasmo coronário, principalmente em aplicações em istmo cavotricuspídeo e istmo mitral,^19^ e lesão renal aguda^20^ nos pacientes com número elevado de aplicações quando se busca o isolamento da parede posterior. Entretanto, algumas estratégias já foram descritas para prevenir essas complicações com o uso de nitrato e hidratação.

Os países europeus vêm usando a nova tecnologia na prática clínica desde 2020, os EUA a partir de fevereiro de 2024 após a aprovação do Food and Drug Administration (FDA), e o PFA encontra-se também disponível no Brasil após a aprovação da Agência Nacional de Vigilância Sanitária (ANVISA) em abril de 2024.

O maior desafio das instituições no Brasil é tornar essas novas tecnologias acessíveis para a população assistida pelo Sistema Único de Saúde (SUS). Até o momento, não existe aprovação pelo SUS das técnicas citadas e usadas amplamente na rede de saúde suplementar. Assim, estudos clínicos avaliando o custo-efetividade das atuais tecnologias se fazem necessários, assim como estudos de viabilidade econômica para sua implantação sistêmica.
