# Supplementary material for: Impacto da Ablação com Campo Pulsado na de Fibrilação Atrial
Source: Arq Bras Cardiol. 2024 Oct 24;121(10):e20240565. [Article in Portuguese] doi: 10.36660/abc.20240565 (PMC11634214; doi:10.36660/abc.20240565)
Supplement: Translation

# Translation: Impact of Pulsed Field Ablation on Atrial Fibrillation

**DOI:** 10.36660/abc.20240565i

**Authors:** Mauricio Scanavacca, Cristiano Pisani

**Affiliations:** 1 São Paulo SP Brazil Instituto do Coração do Hospital das Clínicas da Faculdade de Medicina da Universidade de São Paulo, São Paulo, SP – Brazil

**Keywords:** Atrial Fibrillation, Catheter Ablation, Pulsed Field Ablation, Electroporation

Atrial fibrillation (AF) is the most common cardiac arrhythmia in clinical practice, with an increasing incidence in more elderly patients. It is well known that AF is a substantial risk factor for cardiovascular events, increasing morbidity and mortality among affected patients, in addition to causing reduced physical and cognitive capacity and quality of life.^1-3^ Current treatment of AF is based on modification of aggravating clinical factors, introduction of anticoagulants based on individualized risk-benefit ratio, medications to control heart rate and antiarrhythmics to control rhythm, and electrical cardioversion in patients with persistent AF.^1^

In patients who are refractory or intolerant to medical treatment, catheter ablation with electrical pulmonary vein isolation has been recommended with increasing enthusiasm by international guidelines. In fact, technological adjustments achieved in recent years in point-to-point radiofrequency ablation and balloon cryoablation have promoted greater effectiveness of the procedure and increased safety with both technologies, as demonstrated in numerous recent comparative scientific studies.^4,5^

Radiofrequency and cryoenergy are classified as thermal energies, as their therapeutic effects result from releasing heat during radiofrequency ablation or pronounced cooling during cryoablation. Although they have opposite effects, both conditions cause precise coagulation necrosis of the atrial tissue in contact with the catheter.^6^ The goal of releasing energy around pulmonary veins is to electrically isolate the triggering foci of the pulmonary veins from the left atrium and thus prevent the occurrence of AF. The main challenge over the years has been to achieve a definitive circumferential, transmural scar with satisfactory long-term clinical results.^7,8^

One of the main limitations to the use of these technologies is the precise adjustment of energy delivery. This adjustment must guarantee that the energy released causes the necessary linear atrial, periosteal, contiguous, and transmural lesion, without causing stenosis of the pulmonary veins or reaching adjacent tissues in an undesirable manner, for example, phrenic nerve lesions, when the energy is applied to the right pulmonary veins, or thermal lesions in the esophagus during applications in the antral region or in isolation of the posterior wall of the left atrium.^9,10^

Pulsed field ablation (PFA) is the newest form of energy used for electrical pulmonary vein isolation that creates lesions in the myocardial tissue through irreversible electroporation, with minimal thermal effect on the affected tissues. Biophysical and clinical studies on the adjustment of this energy in electrophysiology began approximately 15 years ago, with clinical results in patients with AF in the past 5 years.^11^

This new form of energy for ablation is characterized by the release of high-energy electrical pulses by the catheter, with an extremely short pulse duration lasting only a few microseconds. These pulses cause the opening of pores in the cell membrane in contact with the catheter electrodes, generating a flood of substances from the tissue fluid inside and their inactivation. Depending on the characteristics of the electrical pulse and the energy of the electrical field, the changes may be transient or permanent, with more intense cell death in specific tissues, an interesting characteristic that shows selectivity in tissue injury.^11^

The equipment that is currently being introduced for clinical use has been tested and adjusted in experimental evaluations and preclinical studies, and it has shown to be very effective in causing irreversible electroporation in atrial tissue, without significantly affecting the pulmonary vein walls, the phrenic nerve juxtaposed to the right pulmonary veins, or the esophagus.^11^

Various clinical trials have demonstrated the efficacy of PFA in patients with drug-refractory paroxysmal AF, with the absence of atrial arrhythmias ranging from 66% to 87% 1 year after pulmonary vein isolation and incidence rates ranging from 0% to 2.5% for severe adverse events.^12-16^ Additionally, they demonstrated shorter procedure times and a quick learning curve. These technical aspects make AF ablation more accessible to the population, involving more fragile patients.

A recently published registry involving 17,642 patients from European centers confirmed the preliminary safety observations, with no reported cases of esophageal lesions, pulmonary vein stenosis, or phrenic paralysis, generating great enthusiasm worldwide.^17^ Another finding from this registry was that the number of complications decreased from the initial 1,700 cases to the subsequent 17,000. Additionally, a recent randomized non-inferiority study demonstrated equivalence in the success of the procedure when comparing the three forms of thermal energy (radiofrequency and cryoablation) and irreversible electroporation.^18^

Despite its safety, the use of this technology has been associated with some specific adverse effects related to this type of energy. One of them is coronary spasm, mainly in applications in the cavotricuspid isthmus and mitral isthmus,^19^ and acute kidney injury^20^ in patients with an elevated number of applications aiming to isolate the posterior wall. Nonetheless, some strategies have already been described to prevent these complications with nitrate use and hydration.

European countries have been using this new technology in clinical practice since 2020. It has been used in the United States since February 2024 after it was approved by the Food and Drug Administration (FDA), and PFA is also available in Brazil after its approval by the Brazilian National Health Surveillance Agency (ANVISA) in April 2024.

The greatest challenge that institutions in Brazil face is to make this new technology accessible to the population treated through the Unified Health System (SUS). To date, the SUS has not approved the techniques discussed herein, which are widely used in the supplementary health network. Therefore, clinical studies evaluating the cost-effectiveness of current technologies are necessary, as are economic feasibility studies for their systemic implementation.
